# Complete genome sequence of *Enterococcus durans* KLDS6.0933, a potential probiotic strain with high cholesterol removal ability

**DOI:** 10.1186/s13099-018-0260-y

**Published:** 2018-07-19

**Authors:** Bailiang Li, Smith Etareri Evivie, Da Jin, Yueyue Meng, Na Li, Fenfen Yan, Guicheng Huo, Fei Liu

**Affiliations:** 1 Key Laboratory of Dairy Science, Ministry of Education, Northeast Agricultural University, Harbin, 150030 People’s Republic of China; 2 Food Science and Nutrition Unit, Department of Animal Science, Faculty of Agriculture, University of Benin, PMB 1154, Benin City, Nigeria; 3 Food College, Northeast Agricultural University, Harbin, 150030 People’s Republic of China

**Keywords:** *Enterococcus durans*, Genome, Probiotic, Stress, l-Tryptophan, Cholesterol removal

## Abstract

**Background:**

*Enterococci* are commensal bacteria in the mammalian gastrointestinal tract which play an important role in the production of various fermented foods. Thus, certain enterococcal strains are commonly used as probiotics to confer health benefits to human and animals. *Enterococcus durans* KLDS6.0933 is a potential probiotic strain with high cholesterol removal ability, which was isolated from traditional naturally fermented cream in Inner Mongolia of China. To better understand the genetic basis of the probiotic properties of this strain, the whole-genome sequence was performed using the PacBio RSII platform.

**Results:**

*Enterococcus durans* KLDS6.0933 contains a circular chromosome of 2,867,028 bp, two plasmids of 163,286 bp and 41,490 bp, respectively. Within the 2704 predicted genes, genes involved with acid, bile and oxidative stress resistance were identified. Bile salt hydrolase (BSH, LIANG_RS13510), a cholesterol removal enzyme identified in the *E. durans* KLDS6.0933 genome is different from that of other *Enterococcus* strains. Furthermore, unlike other *Enterococcus* strains, *E. durans* KLDS 6.0933 can facilitate the complete biosynthesis pathway of l-tryptophan.

**Conclusions:**

In silico analysis confirmed the probiotic properties of *E. durans* KLDS6.0933 and may help us exploit the potential applications of *E. durans* KLDS6.0933 as an industrially important strain.

**Electronic supplementary material:**

The online version of this article (10.1186/s13099-018-0260-y) contains supplementary material, which is available to authorized users.

## Introduction

*Enterococci* are Gram-positive lactic acid bacteria (LAB) and comprise 54 species [1], which are ubiquitously present in the environment, food and the gastrointestinal tracts of diverse hosts. *Enterococci* may have important roles in various fermented food as they contribute to the sensory properties and ripening of sausages or certain cheeses, presumably through proteolysis, lipolysis, and citrate utilization [2, 3]. As a prominent member of normal flora, *Enterococci* play a helpful part in the balance between the gut microbiota and the host.

*Enterococci* are commonly used as probiotics to confer health benefits to human and animals. These bacteria can be used in the treatment of irritable bowel syndrome and antibiotic-associated diarrhea in humans as well as in lowering cholesterol levels or regulating immune system to improve health [4–7]. Likewise, the antioxidant potential of *Enterococci* has been studied [8].

*Enterococcus durans* KLDS6.0933 was originally isolated from traditional naturally cream samples collected in Inner Mongolia of China. It has been demonstrated that *E. durans* KLDS6.0933 had the potential to resist acid and bile salt, and assimilate cholesterol in a recent in vitro study [6]. In order to analyze these characteristics and mine probiotic properties of this strain from genomic insights, the whole-genome sequence of *E. duran*s KLDS6.0933 was carried out and analyzed in silico. Comparison of genomic data from *E. durans* KLDS6.0933 with other *Enterococcus* strains may improve our understanding of the traits of *E. durans* KLDS6.0933.

## Methods

### Strain isolation and DNA extraction

*Enterococcus durans* KLDS6.0933 was isolated from traditional naturally fermented cream in Inner Mongolia of China and was available at the Key Laboratory of Dairy Science (KLDS), Northeast Agricultural University (NEAU), Harbin, China. Before use, *E. durans* KLDS6.0930 was activated through three propagation steps in M17 broth (Oxoid Ltd, Hampshire, UK) at 37 °C for 24 h. The genomic DNA of *E. durans* KLDS6.0933 was extracted using the DNeasy Tissue kit (Qiagen, Germany) following the manufacturer’s instruction.

### Genome sequencing, assembly, and analysis

The quantity and purity of total DNA were determined by 2% agarose gel electrophoresis and a NanoDrop™ spectrophotometer. The whole-genome sequence of *E.* *duran*s KLDS6.0933 was carried out on the single molecule real-time by the Pacbio RSII platform (Pacific Biosciences, USA). A 20 K template library was generated and sequenced by P4-C2 chemistry on two cells. The raw data was obtained as 59,078 pair-end reads (484 MB) with an average read length of 8198 bp. The filtered paired-end reads were de novo assembled by using the hierarchical genome assembly process protocol version 3.0 and polished using Quiver [9]. Gene annotation was determined by Annotation NCBI Prokaryotic Genome Annotation Pipeline [10]. Ribosomal RNA genes were identified using RNAmer 1.2 [11] and tRNA genes were detected using tRNAscan SE v. 2.0 [12]. Functional categories of coding sequences (CDSs) were classified by WebMGA, using RPSBLAST program (applied threshold 1e−5) for clusters of orthologous groups (COG) annotation [13]. The circular genomic map was constructed using CGView Server [14]. Functional annotation was performed with the Kyoto Encyclopedia of Genes and Genomes (KEGG) database using Bi-directional Best Hit method by KAAS [15] for analyzing l-tryptophan biosynthetic pathway. Using the BSH sequence from the complete genomes of representative strains, a phylogenetic tree was constructed using MEGA 7.0 software with the neighbor-joining method [16]. Comparative genomic analysis was performed between *E. durans* KLDS6.0933 and other representative *Enterococcus* strains that are relatively close to *E. durans* KLDS6.0933 based on the phylogenetic tree of 16S rRNA gene.

### Quality assurance

The genomic DNA used for sequencing was isolated from a single colony of the *E. durans* KLDS6.0933. The 16S rRNA gene was sequenced and BLAST was conducted against the NCBI database, then the phylogenetic tree based on the 16S rRNA was constructed by MEGA 7.0 software with the Neighbour-joining method. The result clearly indicated this strain belonged to the species *E. durans* (Additional file 1: Figure S1). In addition, the average nucleotide identity (ANI) of the genomic sequences between *E.* *durans* KLDS6.0933 and *E. durans* ATCC6056 was evaluated by the ANI calculator using the OrthoANIu algorithm at the genomic level. Here, we reported that the value of their ANI was 99.66% (Additional file 1: Table S1).

## Results and discussion

### General features

**Fig. 1:**
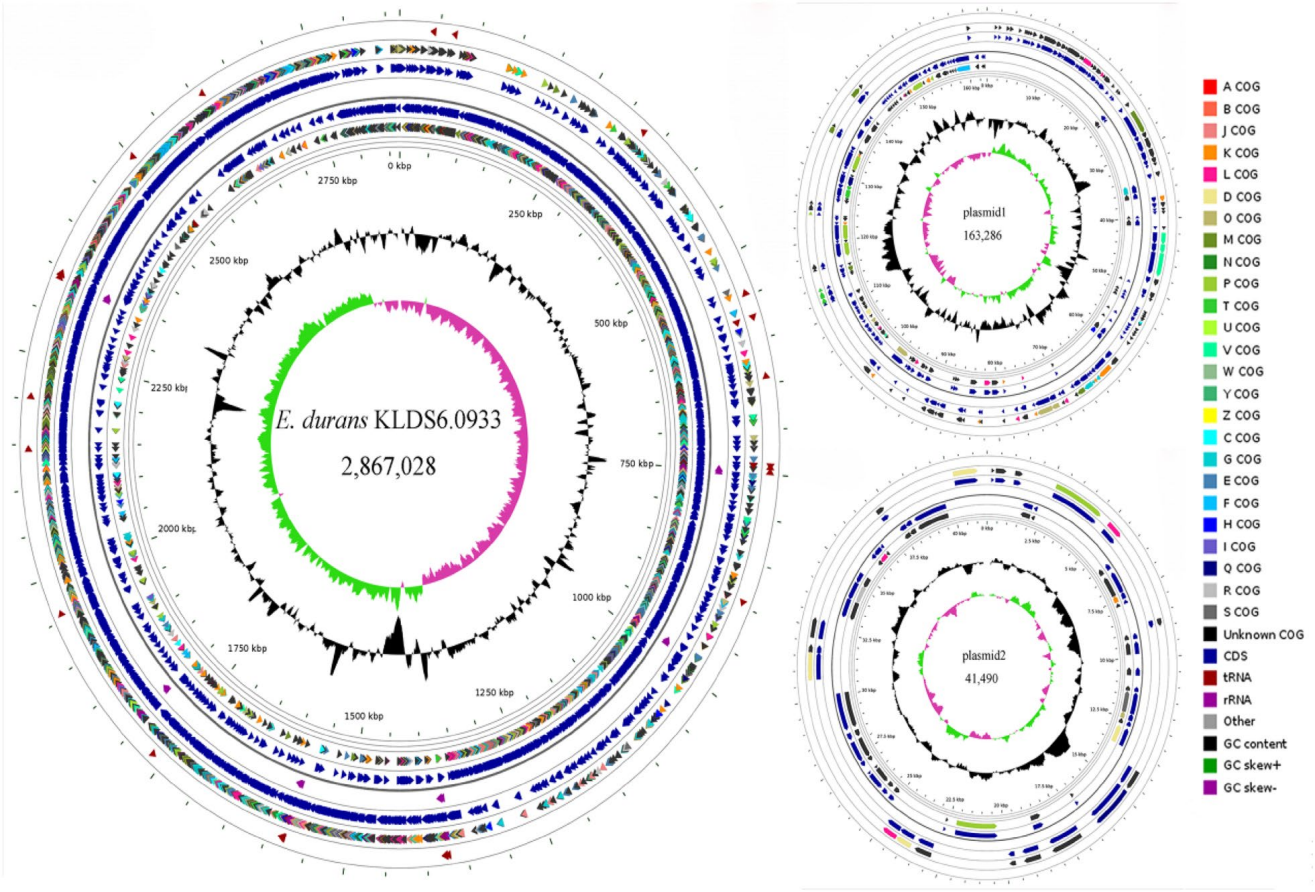
Circular genome map of *Enterococcus durans* KLDS6.0933. From periphery to center: tRNA; Protein coding genes (CDSs) on forward strand colored according to the assigned COG classes; Genes on forward strand; rRNA; Genes on reverse strand; CDSs on reverse strand colored according to the assigned COG classes; Genome position in kbp; GC content; GC skew (G − C)/(G + C)

As shown in Fig. 1, the complete genome of *E. durans* KLDS6.0933 is composed of a 2,867,028 bp chromosome with GC content of 38% and two plasmids—a plasmid of 163,286 bp with a GC content of 35.5% and another plasmid of 41,490 bp with a GC content of 35.3%. Among the 2704 predicted genes, 2393 CDSs, 86 RNAs and 225 pseudogenes were found in the chromosome of *E. durans* KLDS6.0933 (Additional file 1: Table S2). Of the identified CDSs, 2024 genes can be classified into COG classes (Additional file 1: Figure S2). The highest number of genes in this strain was found in the functional groups related to carbohydrate metabolism (212).

### Identification of genes coding stress resistance and cholesterol removal

A recent study reported that *E. durans* KLDS6.0933 was highly tolerant to acid [6]. In order to mine the genetic elements contributing to acid tolerance, proton motive force F_1_F_0_ATPase subunits, Na^+^/H^+^ antiporters, K^+^ uptake transporter and cation-transporting ATPase were analyzed in the genome of *E. durans* KLDS6.0933, *E. durans* KLDS6.0933 possesses these genes by facilitating the counteraction acidic stress (Additional file 1: Table S3). Furthermore, probiotics could increase the intracellular pH via amino acid decarboxylation–antiporter reactions [17], *E. durans* KLDS6.0933 harbors the tyramine cluster which consists of tyrosine decarboxylase, tyrosine tyramine antiporter and tyrosyl-tRNA synthetase gene, which decarboxylate tyrosine to improve the acid tolerance (Additional file 1: Table S3). Another mechanism of acid tolerance is the production of ammonia (alkaline compounds) by the arginine deiminase (ADI) pathway, which includes ADI, ornithine transcarbamylase, carbamate kinase and arginine-ornithine transporter [18]. These genes are presented in *E. durans* KLDS6.0933 (Additional file 1: Table S3).

**Fig. 2:**
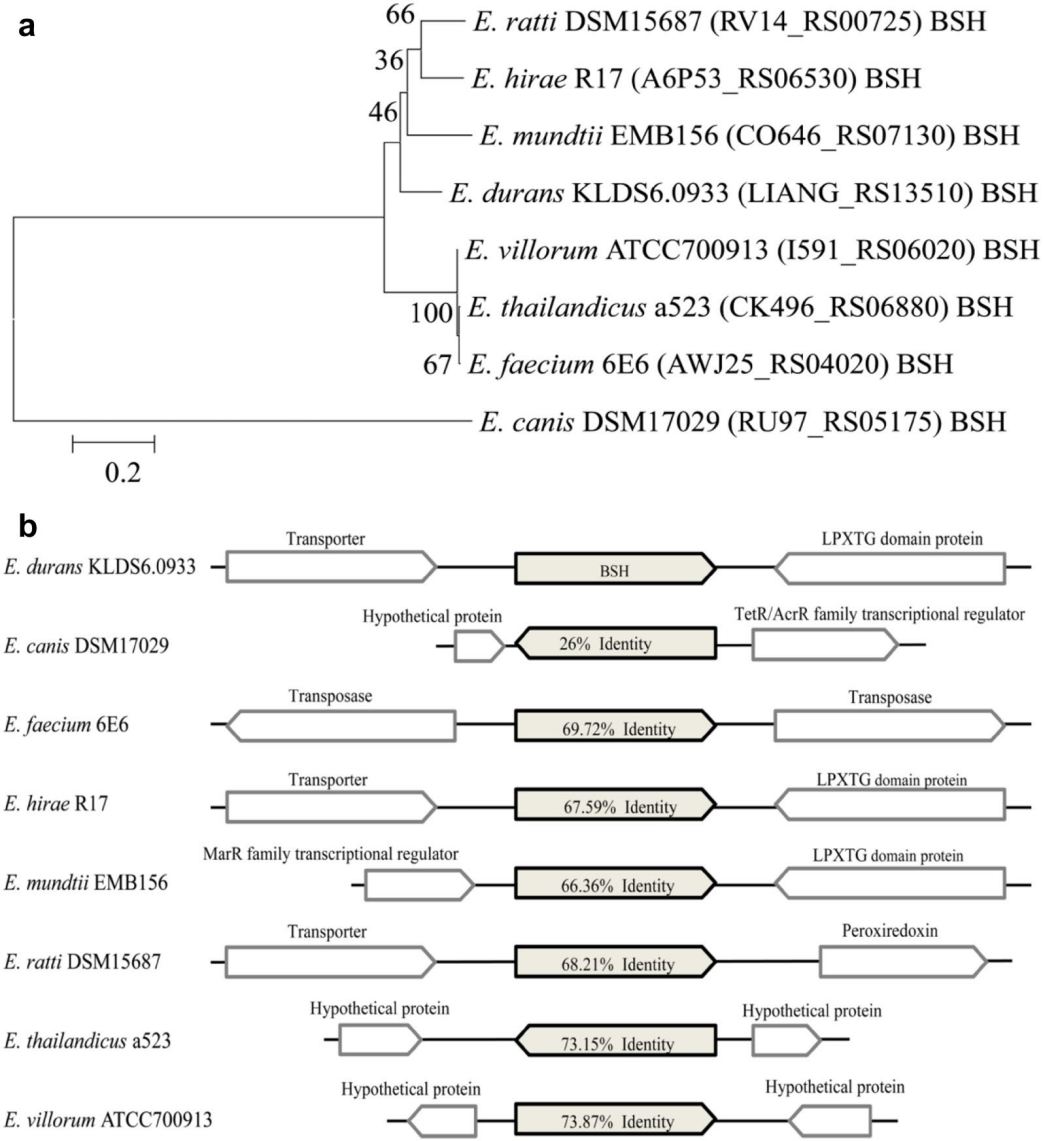
Bioinformatics analysis of bile salt hydrolase (BSH) from *Enterococcus durans* KLDS6.0933. **a** Phylogenetic analysis of BSH. The protein sequences were used to construct a neighbor-joining phylogenetic tree using MEGA 7.0 software with bootstrap replication of 1000. **b** Alignment and comparison of BSH sequences and their contexts using BLASTp

The effect of bile on the growth of *E. durans* KLDS6.0933 showed that this strain had bile tolerance property [6], a gene encoding BSH (LIANG_RS13510), a member of cholylglycine hydrolase family, was identified in the genome of *E. durans* KLDS6.0933, which catalyzes the hydrolysis of glycine- and taurine-conjugated bile salts into amino acid residues and free bile acids [19]. Bile salt deconjugation by this enzyme can lower serum cholesterol level [20]. Supporting this, *E. durans* KLDS6.0933 showed high cholesterol removal ability in our previous study [6]. Phylogenetic relationship among the selected BSH sequences of *E. durans* KLDS6.0933, *E. canis* DSM17029, *E. faecium* 6E6, *E. hirae* R17, *E. mundtii* EMB156, *E. ratti* DSM15687, *E. thailandicus* a523 and *E. villorum* ATCC700913 that had more homology in 16S RNA gene with each other were represented on a neighbor-joining tree, which was constructed using amino acid sequences of BSH with bootstrap replication of 1000 in MEGA 7.0 software. Phylogenetic tree analysis (Fig. 2a) showed that the BSH (LIANG_RS13510) of *E. durans* KLDS 6.0933 is more closely related to that of *E. mundtii* EMB156 than other *Enterococcus* strains. However, they are still evolutionarily distant. In addition, alignment and comparison of BSH sequences and their contexts were studied using BLASTp, as shown in Fig. 2b. We found that BSH sequences showed low sequence identities with that of *E. durans* KLDS6.0933 and the contexts of BSH sequences were different, these imply that BSH is specific to genus and it is significant to further study the relationship between gene structure and enzymatic activity of BSH.

### Identification of genes coding antioxidant system

Oxidative stress occurs when abnormally high levels of reactive oxygen species (ROS) are generated, resulting in nucleic acid, protein and lipid damage [21]. The antioxidant mechanisms of probiotics were associated with enzymatic and non-enzymatic antioxidative system. Among enzymatic antioxidative system, the most conserved oxidative resistance mechanism in the LAB is that oxygen is reduced indirectly to water by coupling of NADH oxidase and NADH peroxidase oxidative [22]. Another important player in resistance to oxidative stress is superoxide dismutase (SOD), which scavenges superoxide anion radicals [21]. Bacteria can also counteract the negative effects of oxidation through catalase, an enzyme catalyzing the detoxification of H_2_O_2_ [23]. Genomic insights into antioxidant activity revealed that the genes encoding NADH oxidase, NADH peroxidase, SOD, catalase and glutathione peroxidase were found in the genome of *E. durans* KLDS6.0933 (Table 1), which are the main components of ROS resistome in the LAB. To cope with oxidative stress, *E. durans* KLDS6.0933 carries diverse genes as shown in Table 1, namely methionine sulfoxide reductase, *S*-methyltransferase, *S*-ribosyl homocysteinase and *S*-adenosylmethionine synthetase, alkyl hydroperoxide reductase and organic hydroperoxide reductase [24].

**Table 1:** Putative genes for antioxidative response in *Enterococcus durans* KLDS6.0933

Encoded protein	Locus tag(s)
NADH oxidase	LIANG_RS12680
NADH peroxidase	LIANG_RS04555
Manganese superoxide dismutase	LIANG_RS10670
Manganese catalase	LIANG_RS08960
Methionine sulfoxide reductase	LIANG_RS06665, LIANG_RS07790, LIANG_RS11590
	LIANG_RS05685
*S*-Adenosylmethionine synthetase	LIANG_RS07580
*S*-Adenosylmethionine ribosyltransferase	LIANG_RS05360
*S*-Ribosyl homocysteinase	LIANG_RS04630
Alkyl hydroperoxide reductase	LIANG_RS00005
Organic hydroperoxide reductase	LIANG_RS13495
Glutathione peroxidase	LIANG_RS04635
Glutathione reductase	LIANG_RS11780
Glutathione-disulfide reductase	LIANG_RS06555
Thioredoxin	LIANG_RS01070
Thioredoxin-disulfide reductase	LIANG_RS02305
Thiol reductase thioredoxin	LIANG_RS02900, LIANG_RS03115, LIANG_RS08005
Redox-sensing transcriptional repressor Rex	LIANG_RS03455, LIANG_RS10045
DNA-binding ferritin-like protein	LIANG_RS06680, LIANG_RS08210
Stress protein	LIANG_RS08010, LIANG_RS08315, LIANG_RS08700
	LIANG_RS00090, LIANG_RS00170, LIANG_RS00205
	LIANG_RS02415, LIANG_RS03580, LIANG_RS05495
	LIANG_RS11215, LIANG_RS02410, LIANG_RS12370

The non-enzymatic antioxidative system has been suggested to be mainly composed of mercapto peptides and regulators. *E. durans* KLDS6.0933 harbors complete glutathione and thioredoxin systems. The presence of glutathione reductase, glutathione-disulfide reductase, thioredoxin, thiol reductase thioredoxin and thioredoxin-disulfide reductase in the genome confers it with antioxidant capacity (Table 1). Regulators and stress proteins contribute to triggering different stress responses to protect against oxidative damage when bacterial cells encounter a specific stress condition [22]. These genes were found in the genome of *E. durans* KLDS6.0933 (Table 1).

### l-Tryptophan biosynthesis pathway

**Fig. 3:**
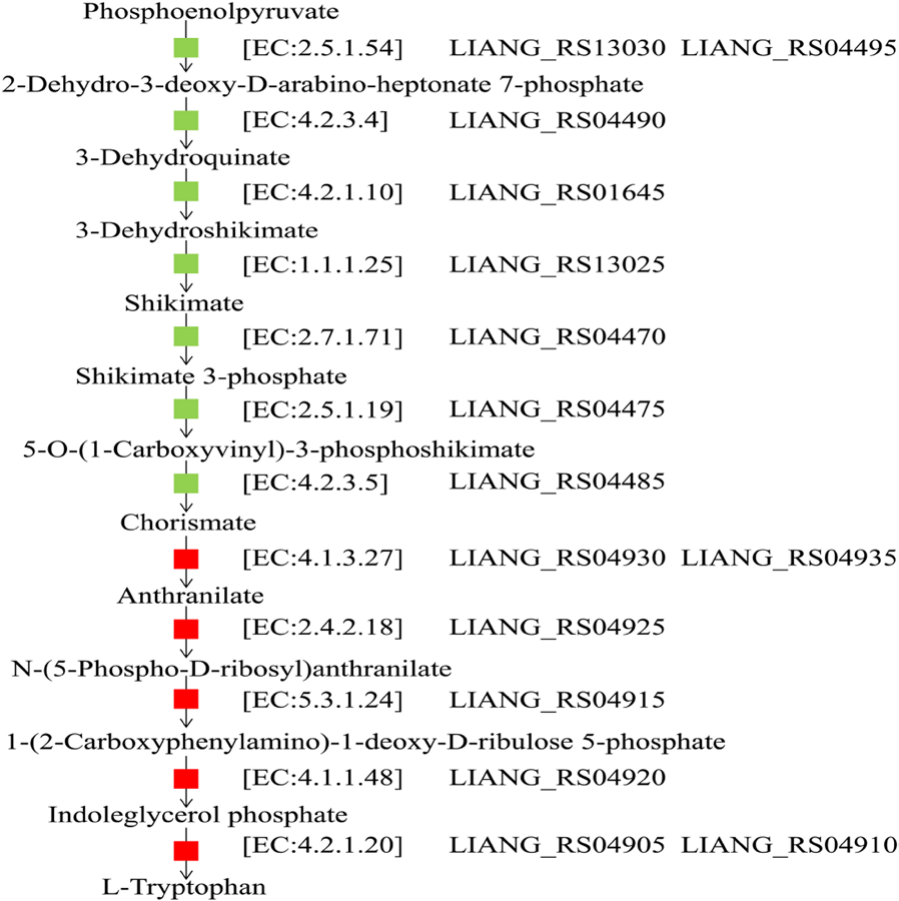
Overview of the l-tryptophan biosynthetic pathway in *Enterococcus durans* KLDS6.0933. Green indicates the presence of the enzyme in all selected *Enterococcus* strains, red indicates the presence of the enzyme only in *E. durans* KLDS6.0933. The gene name of the enzyme in the complete genome of *E. durans* KLDS6.0933 is indicated. The pathway was derived from the Kyoto Encyclopedia of Genes and Genomes (KEGG) pathways

Comparison of the genomes of *E. durans* KLDS6.0933, *E. canis* DSM17029, *E. faecium* 6E6, *E. hirae* R17, *E. mundtii* EMB156, *E. ratti* DSM15687, *E. thailandicus* a523 and *E. villorum* ATCC700913 revealed that only *E. durans* KLDS6.0933 can synthesize l-tryptophan, which is an essential amino acid for humans and other animals and widely used in food, animal feed, and pharmaceutical industries [25, 26]. *E. durans* KLDS6.0933 presents a complete l-tryptophan biosynthetic pathway (Fig. 3) and uses phosphoenolpyruvate as an intermediate, which can be formatted from the pathway of glycolysis. The part of this gene set was also found in other selected *Enterococcus* strains, but these strains lack genes encoding anthranilate synthase, anthranilate phosphoribosyltransferase, phosphoribosylanthranilate isomerase, indole-3-glycerol phosphate synthase and tryptophan synthase, which are essential for producing l-tryptophan from chorismate. Furthermore, the genome of *E. durans* KLDS6.0933 does not carry any genes related to the l-tryptophan degradation pathway, thus indicating that *E. durans* KLDS6.0933 can biosynthesize l-tryptophan de novo.

In conclusion, the complete genome sequence of *E. durans* KLDS6.0933 allows us to better understand the genetic basis of its probiotic potentials. These data will help us explore its potential applications as an important strain in the food industry. However, more in vivo and in vitro researches need to be done to verify the probiotic properties and evaluate its safety status.

## Additional file

**Additional file 1: Figure S1.** Neighbour-joining tree based on the 16S rRNA gene sequences of strain KLDS6.0933 and phylogenetically related *Enterococcus* strains. Bootstrap values based on 1000 resampled datasets are shown at branch nodes. **Figure S2.** Clusters of orthologous groups (COG) functional categories in the complete genome of *Enterococcus durans* KLDS6.0933. **Table S1.** Average nucleotide identity (ANI) of the genomic sequences between *Enterococcus durans* KLDS6.0933 and *Enterococcus durans* ATCC6056. **Table S2.** General genome features of *Enterococcus durans* KLDS6.0933. **Table S3.** Putative genes for acid stress response in *Enterococcus durans* KLDS6.0933.

## Abbreviations

BSH: bile salt hydrolase
LAB: lactic acid bacteria
COG: clusters of orthologous groups
KEGG: Kyoto Encyclopedia of Genes and Genomes
CDSs: coding sequences
ANI: average nucleotide identity
ADI: arginine deiminase
ROS: reactive oxygen species
