# Supplementary material for: Complete genome sequence of Enterococcus durans KLDS6.0933, a potential probiotic strain with high cholesterol removal ability
Source: Gut Pathog. 2018 Jul 19;10:32. doi: 10.1186/s13099-018-0260-y (PMC6052589; doi:10.1186/s13099-018-0260-y)
Supplement: Supplementary file 1 — Additional file 1: Figure S1. Neighbour-joining tree based on the 16S rRNA gene sequences of strain KLDS6.0933 and phylogenetically related Enterococcus strains. Bootstrap values based on 1000 resampled datasets are shown at branch nodes. Figure S2. Clusters of orthologous groups (COG) functional categories in the complete genome of Enterococcus durans KLDS6.0933. Table S1. Average nucleotide identity (ANI) of the genomic sequences between Enterococcus durans KLDS6.0933 and Enterococcus durans ATCC6056. Table S2. General genome features of Enterococcus durans KLDS6.0933. Table S3. Putative genes for acid stress response in Enterococcus durans KLDS6.0933. [file 13099_2018_260_MOESM1_ESM.docx]

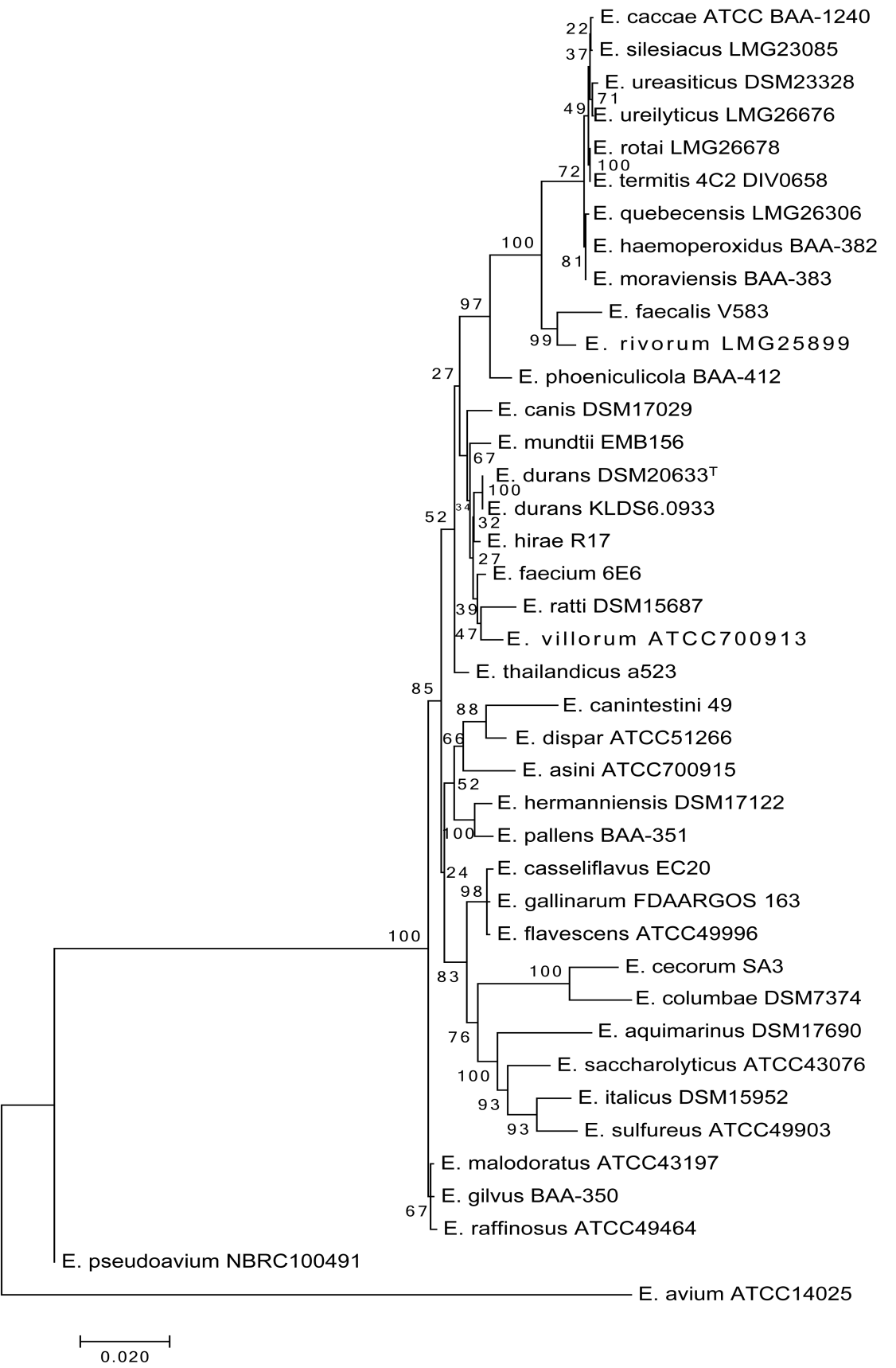


**Figure S****1** Neighbour-joining tree based on the 16S rRNA gene sequences of strain KLDS6.0933 and phylogenetically related *Enterococcus* strains**.** Bootstrap values based on 1000 resampled datasets are shown at branch nodes.


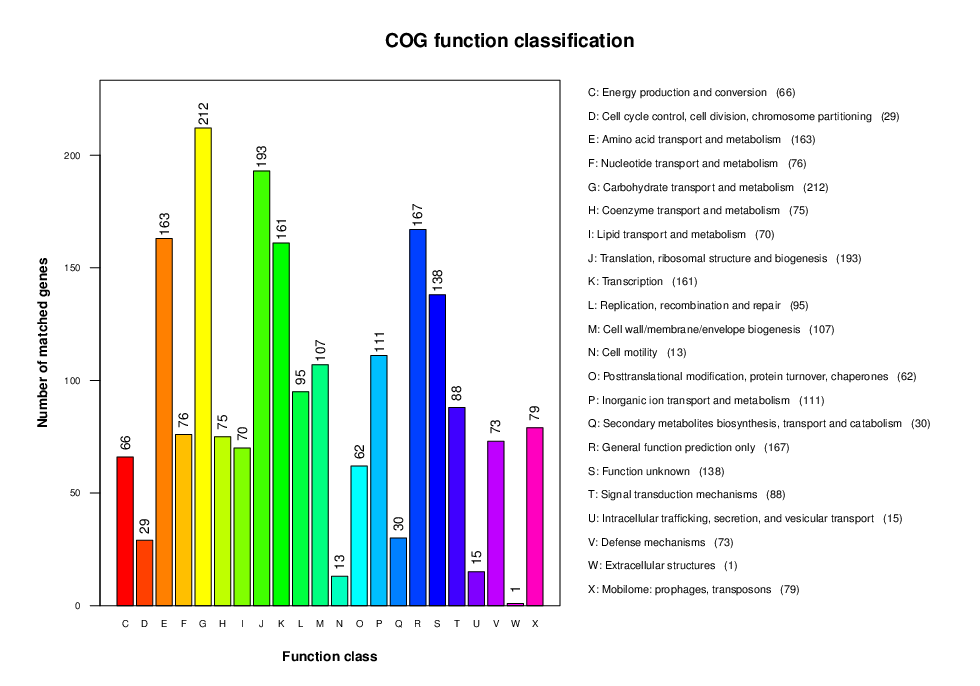


**Figure S2** Clusters of Orthologous Groups (COG) functional categories in the complete genome of *Enterococcus durans* KLDS6.0933.

**Table S1** Average nucleotide identity (ANI) of the genomic sequences between *Enterococcus durans* KLDS6.0933 and [*Enterococcus*](https://www.ncbi.nlm.nih.gov/genome/11519?genome_assembly_id=245829)*durans* ATCC6056.

| Metric | Value |
| --- | --- |
| OrthoANIu value (%) | 99.66 |
| *E. durans* KLDS6.0933 genome length (bp) | 2,866,200 |
| *E. durans* ATCC6056 genome length (bp) | 3,165,060 |
| Average aligned length (bp) | 1,996,544 |
| *E. durans* KLDS6.0933 genome coverage (%) | 69.66 |
| *E. durans* ATCC6056 genome coverage (%) | 63.08 |

**Table S2** General genome features of *Enterococcus durans* KLDS6.0933.

| Feature | Chromosome | plasmid 1 | plasmid 2 |
| --- | --- | --- | --- |
| Size [bp] | 2,867,028 | 163,286 | 41,490 |
| GC content [%] | 38.0% | 35.5% | 35.3% |
| Predicted genes | 2,704 | 182 | 51 |
| Protein coding genes (CDSs) | 2,393 | 155 | 47 |
| Pseudogenes | 225 | 27 | 4 |
| rRNA operons | 6 | 0 | 0 |
| tRNAs | 68 | 0 | 0 |
| ncRNA | 0 | 0 | 0 |
| Frameshifted Genes | 188 | 0 | 0 |
| GenBank accession | CP012366.1 | CP012367.1 | CP012368.1 |

**Table S3** Putative genes for acid stress response in *Enterococcus durans* KLDS6.0933.

| Encoded protein | Locus tag(s) |
| --- | --- |
| F_1_F_0_ATPase subunits | LIANG_RS02605, LIANG_RS02610 |
|  | LIANG_RS02615, LIANG_RS02620 |
|  | LIANG_RS02625, LIANG_RS02630 |
|  | LIANG_RS02635, LIANG_RS02640 |
| Na+/H+ antiporters | LIANG_RS05115, LIANG_RS11550, LIANG_RS09745 |
| K+ uptake transporter | LIANG_RS05525, LIANG_RS10640, LIANG_RS12530 |
| cation-transporting ATPase | LIANG_RS01945, LIANG_RS01950 |
|  | LIANG_RS01955, LIANG_RS09715 |
| tyrosine decarboxylase | LIANG_RS05125 |
| tyrosine tyramine antiporter | LIANG_RS05120 |
| tyrosyl-tRNA synthetase | LIANG_RS05130 |
| arginine deiminase | LIANG_RS01395 |
| ornithine transcarbamylase | LIANG_RS01390 |
| carbamate kinase | LIANG_RS01385 |
| arginine-ornithine transporter | LIANG_RS09810 |
